# Infantile Mortality

**Published:** 1905-10-07

**Authors:** 


### Infantile Mortality.

Dr. J. T. C. Nash, the Medical Officer of Health for Southend, contributes to the October number of the "Journal of the Royal Sanitary Institute" an important and suggestive paper upon "The Waste of Infant Life," in which he calls attention to the circumstance that, although many of the factors to which this waste is commonly attributed, such as overcrowding, want of ventilation and of sunlight, maternal industries, improper methods of feeding, proprietary so-called "infants' foods," the intemperance of parents, and so forth, are mostly in uninterrupted operation throughout the year, the waste of life itself is much increased at certain seasons, especially in late summer and early autumn, when its immediate cause is the prevalence of infantile diarrhœa. The late Dr. Ballard, of the Local Government Board, pointed out, many years ago, that the seasonal incidence of diarrhœa usually coincides with a time when the ground temperature rises to 56 degrees F. or more, at a depth of four feet below the surface, and he assumed that this temperature was necessary for, or favourable to, the development of some micro-organism capable of producing diarrhœa, and existing in organically polluted soil. Dr. Nash, while admitting the facts, is not satisfied with the inference; and expresses his belief that the nature of the connection between the summer temperature and the prevalence of diarrhœa is chiefly that the former is favourable to the multiplication of the ordinary housefly, *musca domesticà*, and that this creature is largely responsible for the pollution of milk, one of its favourite foods, by the mechanical conveyance to it of noxious matters derived from dung, decaying refuse, and other matters upon which flies are wont to congregate. He quotes an observation which he published in 1903, to the effect that, during the preceding summer, although the deep temperature required by Dr. Ballard's hypothesis was maintained for some weeks, there was not the usual incidence of infantile diarrhœa, and that this immunity was associated with a remarkable scarcity of house flies. In the early part of September flies became fairly prevalent in Southend, and diarrhœa, which had been conspicuous for its absence, speedily occasioned thirteen deaths. Then came a spell of cold weather, the flies rapidly diminished in number, and no more deaths from diarrhœa were recorded.

It is impossible, of course, safely to draw general conclusions from a single observation, however pertinent to the question at issue it may appear; but it cannot be denied that the observations of Dr. Nash may be of great practical value, or that his position, that milk is even more liable to be dangerously polluted after than before delivery to the purchaser, is completely in consonance with ordinary experience of methods of domestic storage in houses of the poorer class. It would not be difficult to institute a series of experiments, the results of which would be of unquestionable interest and importance. A comparative examination of samples of milk which had been exposed to the visits of flies in the habitation of the purchaser with control samples in like positions which had been protected from them, and instituted at seasons when flies were abundant, and also at seasons when they were absent or inactive, would at least afford a definite indication of the part presumably taken by the insects as conveyers of bacteria, and also of the condition of the milk before and after it had been exposed to their depredations. The milk jug, moreover, is a common grave of flies who fall victims to their own greediness; and, as there is high authority for the assertion that their dead bodies cause "the ointment of the apothecary to send forth a stinking savour," it is more than probable that, if sufficient time were given, they would be equally effective in the case of milk. Apart from seasonal changes which may determine their scarcity or abundance, it must not be forgotten that flies are always attracted to a dirty house, and that their presence or absence, other things being equal, may safely be regarded as an indication of domestic cleanliness or slatternliness, as the case may be. It is probable that the first glance of a surgeon, in considering the fitness or unfitness of a room in a private house for an operation, would be directed towards the window, with a view to the presence or absence of flies. If cleanliness prevail in corners, flies will be driven to seek a more congenial habitat elsewhere; and, in like manner, if public scavenging be effective, their numbers in any locality will be inconsiderable. There will be no need to invoke the assistance of Beelzebub, the fly god, the averter of insects, if public and private authorities can only be induced to perform their respective duties in an efficient and creditable manner.

